# A High-Precision Fully Integrated Hall-Effect Angle Sensor with 0.087° Noise Floor in 0.35 μm CMOS Technology

**DOI:** 10.3390/s26134284

**Published:** 2026-07-06

**Authors:** Zhenzhong Yuan, Yang Zhao, Yingdan Jiang, Xiangyi Kong

**Affiliations:** 1 School of Integrated Circuits, Shanghai Jiao Tong University, Shanghai 200240, China; yngsky@yeah.net; 2 The 58th Research Institute of China Electronics Technology Group Corporation, Wuxi 214063, China; ydjiang@163.com (Y.J.);

**Keywords:** Hall-effect, angle sensor, high accuracy

## Abstract

Hall-effect sensors are pervasive in magnetic-field measurement applications, including current sensing and position detection, owing to their excellent compatibility with standard CMOS processes. However, the inherent offset and temperature drift of silicon-based Hall elements remain a paramount obstacle to achieve high precision. This paper presents a fully integrated angle sensor chip that addresses this challenge. Implemented in a 0.35 μm CMOS process, the sensor incorporates four cross-shaped Hall elements arranged in an orthogonal array as a non-contact Hall-permanent magnet configuration, which enables absolute angular encoding across a full 0–360° range. Experimental characterisation demonstrates a low noise floor of 0.087° (3σ), validating the effectiveness of the proposed architecture for high-accuracy angular measurement.

## 1. Introduction

In the contemporary era of intelligent systems, sensors serve as the critical interface bridging the physical and digital realms, rendering them indispensable components in industrial automation, modern communication infrastructures, and everyday smart devices. Among these, angular position sensors—which measure the relative displacement between stationary (stator) and rotating (rotor) components—are of particular importance for high-performance applications, including motor control, robotic joints, and automotive steering systems.

State-of-the-art angular measurement is accomplished through several modalities: resolvers [1], inductive sensors [2,3], optical encoders [4], and magnetic encoders [5]. However, the first three approaches are encumbered by inherent drawbacks that preclude their adoption in miniaturised systems, including high manufacturing costs, susceptibility to electromagnetic interference, and considerable system-level complexity. In stark contrast, magnetic encoders based on Hall-effect elements have garnered substantial attention and found widespread application in integrated sensing systems. Their ascendancy stems from superior noise immunity, a high degree of integration, and excellent compatibility with standard CMOS processes. Against this backdrop, the design and optimisation of magnetic angular sensors have emerged as a critical area of research, driven by ever-increasing demands for enhanced measurement accuracy, operational reliability, and system-level integration. To this end, this work presents a fully integrated Hall-effect angle sensor designed to address the existing limitations in precision and compactness. The core architecture of a Hall-effect sensor is implemented through the synergistic integration of a differential Hall-element array and an on-chip signal conditioning chain. Two quadrature-phase magnetic induction signals are utilised to achieve 360° angle computation with no blind spots, and a dedicated CORDIC (Coordinate Rotation Digital Computer) module is embedded to provide real-time high-resolution digital angle outputs.

## 2. Circuit Design

### 2.1. Hall Sensor Circuit Design

**Figure 1:**
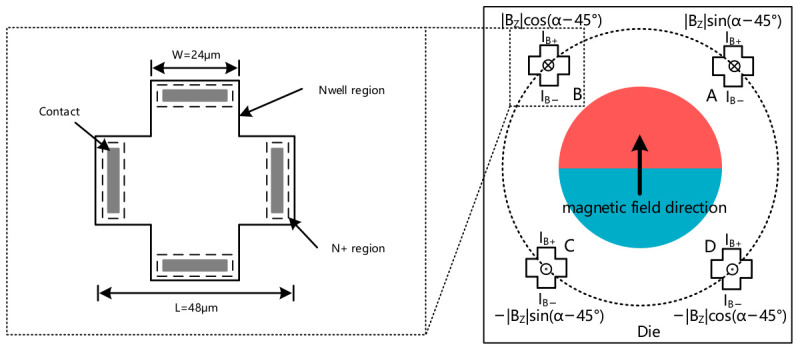
Design and layout of Hall sensor.

The Hall-effect sensor presented in this study was fabricated using a standard 0.35 μm CMOS N-well process. A schematic illustration of the microstructure is provided in Figure 1, along with critical dimensions [6,7]. The active region of the device features a symmetric cross-shaped architecture. Compared to conventional square Hall plates, a performance of enhanced sensitivity and reduced offset can be provided with a given device area [8,9].

Hall devices operate on the bias conditions of either voltage or current. Previous studies reported that an eight-phase spinning-current technique combining voltage bias with current readout can substantially improve offset and sensitivity performance [10,11]. In this work, to reduce circuit complexity while maintaining balanced device performance, a four-phase spinning-current scheme with constant-current bias was adopted. Some studies have fabricated Hall sensors using an inverted triangular structure. Although this approach enhances sensor sensitivity, it suffers from high cost and complex processing [12]. Although this method can improve the sensitivity of the Hall sensor, its adoption of MEMS micromachining (TMAH etching) and CMOS processes leads to a complex fabrication process, and it still requires the spinning-current method to suppress the offset voltage of the Hall device. Further developing an angle sensor based on such an inverted-pyramid-structured Hall sensor will inevitably increase the complexity of circuit development. The Hall voltage is extracted from the two remaining terminals of each device and exhibits a linear dependence on the out-of-plane magnetic field component. Angular measurement is subsequently realised through the post-processing of the four Hall voltage signals [13,14].

To enable absolute angular position measurement, four Hall elements were arranged concentrically with a shared centre and a radius of 1200 μm. This configuration allows simultaneous detection of the axial magnetic field component (B_Z_) generated by a permanent magnet. Under constant-current bias and an applied magnetic field, the Hall voltage generated by each element satisfies

V_H_ = S_I_I_B_|B_Z_| + V_Hoff_ (1)

(2) $$s_{I}=\frac{G\mu_{H}}{q{\mu_{n}N}_{D}t_{eff}}$$

where S_I_ represents the current-related sensitivity of the cross-shaped Hall element; G is the geometric factor of the Hall device, which is directly related to the shape of the Hall device; μ_H_ is the Hall mobility; μ_n_ is the electron mobility; N_D_ is the doping concentration of the N-well in the semiconductor process; teff is the effective thickness of the N-well; and q is the elementary charge.

The expression for the geometric factor G of the Hall device can be approximately expressed as

(3) $$G\approx 1-5.0267\frac{\theta_{H}}{tan(\theta_{H})}e^{\frac{-\pi L}{2W}}$$

θ_H_ is the Hall angle, which is the angle between the direction of the resultant electric field and the direction of the current. Given a fixed fabrication process for the Hall sensor, G depends solely on W and L.

B_Z_ denotes the equivalent magnetic flux density perpendicular to the sensor plane. The in-plane field components (B_X_ and B_Y_) are not considered in this analysis, as their contribution to the sensor output was determined to be negligible under the operating conditions employed [15].

In this work, the Hall elements were designed with a length of 48 μm and a width of 24 μm. Characterisation results indicate that the Hall sensor achieves a sensitivity of 37.6 V/(A·T) [16]. All four Hall elements share the same excitation direction. Taking the configuration where the bias current flows vertically (from top to bottom) through the sensor as an example, the Hall voltages generated by the four elements can be expressed as

V_HA_ = S_I_I_B_|B_Z_|sin(α − 45°) + V_Hoffset_ (4)

V_HB_ = S_I_I_B_|B_Z_|cos(α − 45°) + V_Hoffset_ (5)

V_HC_ = −S_I_I_B_|B_Z_|sin(α − 45°) + V_Hoffset_ (6)

V_HD_ = −S_I_I_B_|B_Z_|cos(α − 45°) + V_Hoffset_ (7)

The offset voltage of Hall devices is intrinsically linked to fabrication process variations and can be attributed to several factors. First, geometrical asymmetries in the electrode structure—such as alignment deviations during manufacturing and non-ideal symmetry in electrode dimensions—contribute significantly to offset. Second, inhomogeneities in material properties, including spatial variations in resistivity within the semiconductor material, lead to imbalanced current density distributions that adversely affect Hall output characteristics. Third, fabrication-induced structural defects, such as geometric distortions introduced during lithography, etching, and other micro-machining processes, can substantially exacerbate offset voltage generation. To mitigate the impact of offset voltage and realise more accurate angular position measurement, further signal processing of the Hall voltages by the readout circuit is required [17].

### 2.2. Design of Hall Sensor Signal Conditioning Circuit

**Figure 2:**
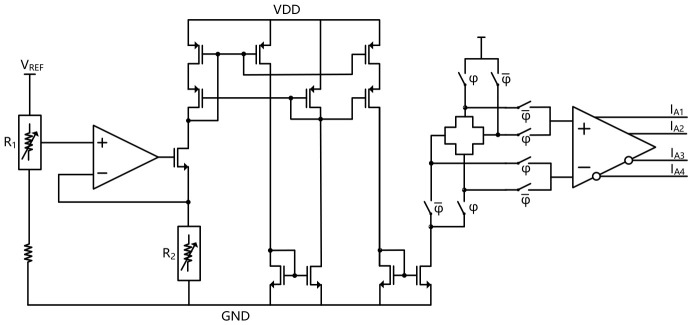
Hall sensor excitation circuit design.

The current excitation circuitry and preamplifier configuration for the Hall sensors are illustrated in Figure 2. To suppress offset voltage arising from fabrication process variations, a spinning-current excitation technique is employed in this study. This approach reduces the residual offset by an order of magnitude, thereby improving the magnetic field measurement accuracy of the Hall sensor to within ±0.3 mT [18]. Resistors R1 and R2 in the circuit are implemented as 8-bit binary-weighted resistor arrays controlled via an SPI digital interface. These programmable resistors compensate for sensitivity drift induced by on-chip gradient stress or process doping variations. In the excitation circuit, the current mirror section employs a PMOS cascode structure, which offers advantages in low-supply-voltage applications [19].

**Figure 3:**
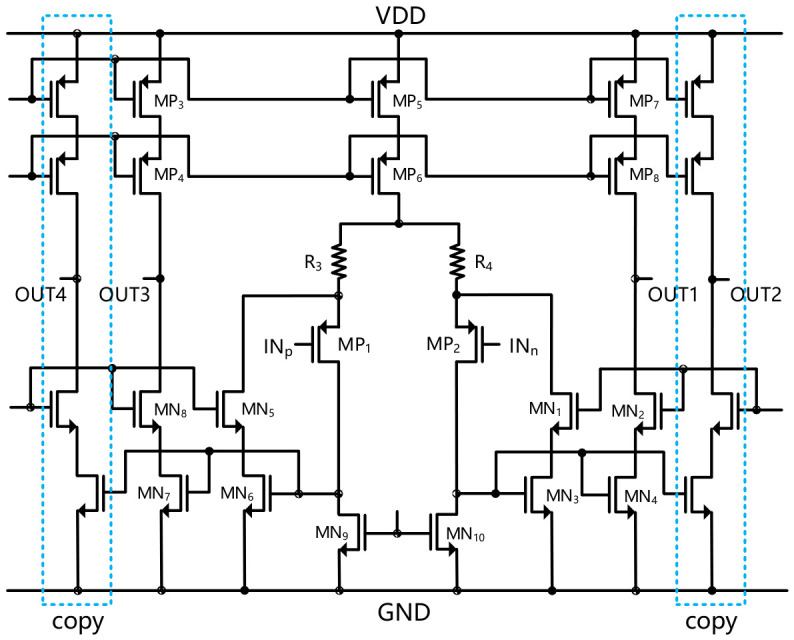
Preamplifier design.

Due to the inherent operating principles of Hall-effect sensors, their output voltage signals are typically weak—on the order of millivolts—and are susceptible to environmental factors such as temperature fluctuations and electromagnetic interference, which can degrade signal quality in practical applications. To achieve high-precision amplification of the Hall voltage, this work employs a fully differential amplification architecture for the preamplifier circuit, as illustrated in Figure 3. The preamplifier is implemented as a two-stage cascaded design. The first stage consists of a fully differential operational amplifier employing source degeneration, while the second stage utilises a telescopic cascode amplifier [20]. In the first stage, source degeneration resistors R3 and R4 are introduced to effectively stabilise the input transconductance, thereby enhancing linearity and gain consistency across varying operating conditions. Although the introduction of the source-degeneration resistor introduces additional noise, its noise contribution at the input is much smaller than that of the Hall sensor itself. Transistors MN1–MN4 and MN5–MN8 similarly form source-degenerated configurations, further improving the linearity of the preamplifier. This design reflects a deliberate trade-off between gain and linearity—a moderate reduction in gain is accepted in exchange for superior linear response and enhanced system stability. This trade-off is critical for preventing linear distortion during Hall voltage amplification and for avoiding output saturation in the operational amplifier. To facilitate subsequent processing of the Hall voltage signals for angular position measurement, the differential output of the operational amplifier is replicated as follows:

V_OUT1 = V_OUT2(8)

V_OUT3 = V_OUT4(9)

The gain of the fully differential amplifier is thus

(10) $$A_{v}\approx g_{mp1}\left[\left(r_{op1}+R_{3}\right)||r_{on9}\right]g_{mp6}\left[\left(r_{on6}g_{mn5}r_{on5}\right)||\left(r_{op3}g_{mp4}r_{op3}\right)\right]$$

### 2.3. Design of Angle Signal Processing Circuit

**Figure 4:**
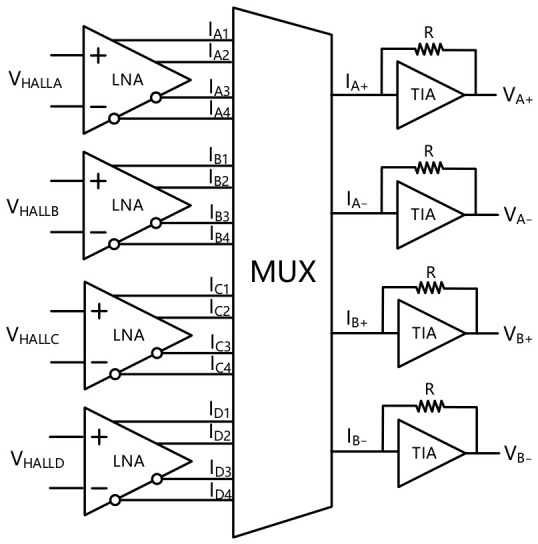
Design of signal angle conditioning for Hall sensor.

To suppress offset voltage induced by process variations and temperature gradients, while simultaneously obtaining electrical signals with high linearity and low noise that are proportional to the angular position of the permanent magnet, this work proposes a signal chain architecture based on ‘current-domain Hall signal processing with transimpedance aggregation.’ A schematic of this architecture is presented in Figure 4. This architecture first converts the four differential Hall voltages—with phase differences of 90° relative to one another—into current signals via transconductance amplifiers. Subsequently, a CMOS multiplexer performs vector summation of these current signals [21]. This operation effectively achieves automatic averaging and cancellation of offset components in the charge domain, retaining only the effective signals as orthogonal components proportional to sinα and cosα. By leveraging signal processing in the current domain, this architecture introduces negligible additional thermal noise and voltage offset during the summation process, thereby enabling more precise processing of the Hall signals by the readout circuitry.

By controlling the selection switches of the multiplexer (MUX), the output signals from the four Hall sensors are processed according to the following operational relationships:

I_A+_ = I_A1_ + I_B1_ + I_C3_+ I_D3_ (11)

I_A−_ = I_A3_ + I_B3_ + I_C1_+ I_D1_ (12)

I_B+_ = I_A4_ + I_B2_ + I_C4_+ I_D2_ (13)

I_B−_ = I_A2_ + I_B4_ + I_C2_+ I_D4_ (14)

Then,

ΔI_A_ = I_A+_ − I_A−_ = Gm(V_HA_ +V_HB_ − V_HC_ − V_HD_) (15)

ΔI_B_ = I_B+_ − I_B−_ = Gm(−V_HA_ +V_HB_ +V_HC_ − V_HD_) (16)

Rewriting ΔI_A_ and ΔI_B_ based on Equations (4)–(7):

ΔI_A_ = GmS_I_I_B_|B_Z_|[2sin(α − 45°) + 2cos(α − 45°)] (17)

ΔI_B_ = GmS_I_I_B_|B_Z_|[−2sin(α − 45°) + 2cos(α − 45°)] (18)

The resulting current signals (ΔI_A_ and ΔI_B_) are subsequently converted into voltage signals (Δ*V_A_* and Δ*V_B_*) via a transimpedance amplifier. The ratio of these two voltage signals is then taken. After further simplification, this ratio yields

(19) $$\frac{\Delta V_{A}}{\Delta V_{B}}=\tan\alpha$$

Following the aforementioned current-domain Hall signal processing chain, low-noise orthogonal signals sinα and cosα—exhibiting a strict quadrature relationship with the rotor angular position α—are obtained. These quadrature signals are digitised by a sigma-delta ADC and subsequently fed directly into a digital CORDIC (Coordinate Rotation Digital Computer) core [22], which performs the arctangent computation to enable real-time output of the absolute angle value over the full 0–360° range.

This study demonstrates notable advantages in the following three aspects:

1. Self-Cancellation of Hall Signal Offset

The vector summation of four-phase Hall current signals effectively cancels both the inherent offset voltage of the Hall sensors and offset variations induced by temperature fluctuations. This significantly enhances angular measurement accuracy.

2. Additional Gain Enhancement without Noise Penalty

During time-domain summation of the four-phase Hall currents, a two-fold gain enhancement is achieved using only MUX cross-switching, without cascading any additional gain amplifiers. Consequently, no new thermal noise or phase delay is introduced. The signal-to-noise ratio (SNR) is improved by 6 dB compared to voltage-domain amplification schemes.

3. Absolute Angle Compensation

Due to packaging constraints, a fixed positional offset exists between the physical locations of the four Hall sensors and the absolute 0° reference (12 o’clock direction). Through the vector summation method of the four-phase Hall current signals, this work successfully remaps the absolute 0° reference to the 12 o’clock direction. This approach maintains consistency between the wafer layout and system zero-position without requiring additional digital processing units, thereby conserving design resources effectively.

## 3. Simulation and Results

**Figure 5:**
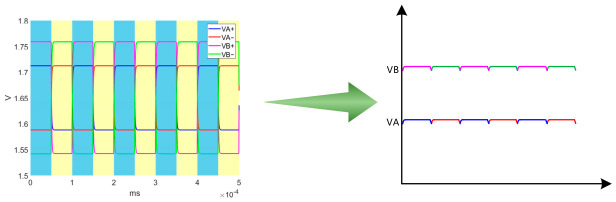
ADC input terminal analogue signal simulation (steady state after circuit power-on) and digital circuit pre-processing results.

Simulated waveforms of ΔV_A_ and ΔV_B_ are presented in Figure 5 (exemplified by a 30° angle). The magnetic field signal, after passing through the Hall sensors and signal conditioning circuitry, is converted into linearly correlated square-wave signals. The peak-to-peak amplitude of these square-wave signals reflects the instantaneous magnetic field strength. Notably, ΔV_A_ and ΔV_B_ are directly digitised by the sigma-delta ADC [23], eliminating one analogue demodulation stage and thereby avoiding the introduction of additional clock noise. A second-order, 1-bit quantiser sigma-delta ADC architecture was selected for this design, achieving an effective number of bits (ENOB) of 14.9 bits.

The quantised digital codes from the ADC undergo digital pre-processing prior to final angle calculation. First, the differential square-wave signals are synchronously demodulated to extract the DC components, with the demodulation phase strictly locked to the Hall sensor excitation signal. Next, high-order digital filtering is applied to suppress high-frequency interference, including clock noise. Finally, a 16-stage iterative CORDIC algorithm performs the arctangent computation, yielding high-precision angular values.

**Figure 6:**
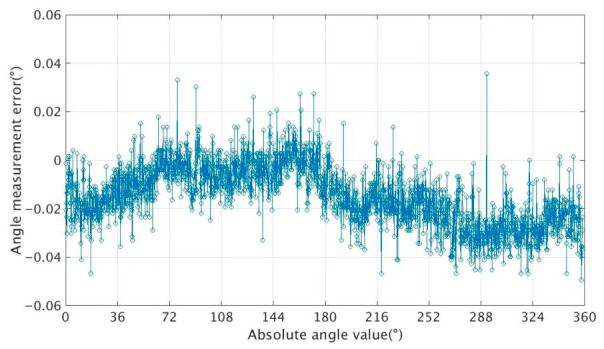
Simulated value of the angular measurement error.

During the simulation phase, a Hall device model was first established using COMSOL Multiphysics software 5.6. The obtained Hall voltage parameters were then used to construct a circuit model in the Virtuoso environment via the Verilog-A language. In the digital circuit, the CORDIC algorithm was employed to perform the arctangent operation. The simulation results of the angular measurement error are presented in Figure 6. The simulated angular measurement error (INL) is less than ±0.05° over the full angular range (0–360°).

Although the analogue front end employs multiple techniques—such as the spinning-current method, current-domain Hall signal conditioning with a transimpedance aggregation architecture, and a 16-bit high-precision ADC—to suppress nonlinear factors such as system offset voltage, residual second-order nonlinearity and temperature drift remain in the signal conditioning circuitry due to wafer stress gradients, package stress relief, and temperature gradients, limiting further improvement in angular resolution. To address this, the digital section in this study incorporates an embedded e-Fuse trim array. During the testing phase, gain error, zero-angle offset, and first-order temperature drift coefficients are extracted via optical encoder calibration, and the compensation coefficients are subsequently programmed into the e-Fuse. Additionally, the excitation current of the Hall sensors can be trimmed via resistors R1 and R2 as shown in Figure 2 to ensure consistency in Hall sensor. This approach effectively enhances absolute angle detection accuracy.

To address this, an embedded e-Fuse trimming array is incorporated in the digital domain. During the testing phase, gain errors, zero-angle offset, and first-order temperature drift coefficients are characterised and extracted using an optical encoder as a reference. The corresponding trimming codes are then programmed into the e-Fuse memory, substantially improving absolute angle detection accuracy. The zero-degree calibration procedure is as follows: in the digital circuit, the calculated angle value is first subtracted by the angle value stored in the zero-position register (the value in this register is controlled by the e-Fuse, with a default value of 0). After writing the uncalibrated angle value into the zero-position register, the angle value read from the SPI interface after subtraction becomes the zero-calibrated angle value.

**Figure 7:**
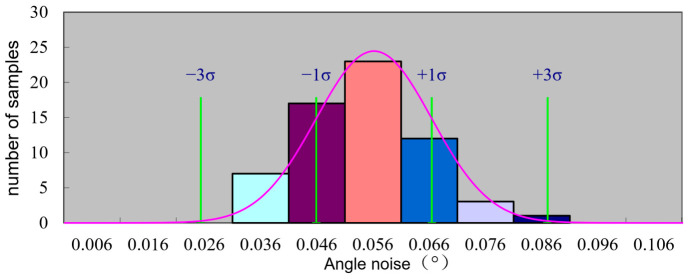
Angle measurement noise.

The noise test of the angle sensor chip was conducted in a magnetically shielded room. During the test, a fixed-angle noise test was performed on 63 angle sensor chips (calibrated) from the same batch. The measured static angle noise, expressed as a 3σ value, was 0.087 deg (3σ). The test results are shown in Figure 7.

**Figure 8:**
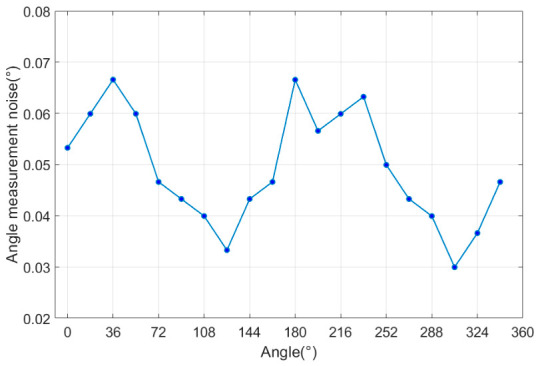
Noise test results under 0–360° full-angle scanning conditions.

The measured angular noise over the full angular range is shown in Figure 8. The angular measurement noise varies with the measured angle, which is mainly attributed to the nonlinear noise gain during the angle calculation process. When the angle is derived from the orthogonal Hall signals via the arctangent function, the rate of signal change is the lowest near the signal zero-crossing points (45° and 225° in this work). Consequently, a voltage noise of fixed amplitude induces the largest angular jitter at these positions. Furthermore, non-ideal characteristics of the Hall sensor (e.g., offset) and the quantisation error of the CORDIC algorithm in different angular regions further exacerbate this dependency. A comparison of key parameters is shown in Table 1.

At a fixed angle, the noise performance of the angle sensor significantly deteriorates at 95 °C. The test results of the angular measurement noise as a function of temperature are presented in Table 2.

**Figure 9:**
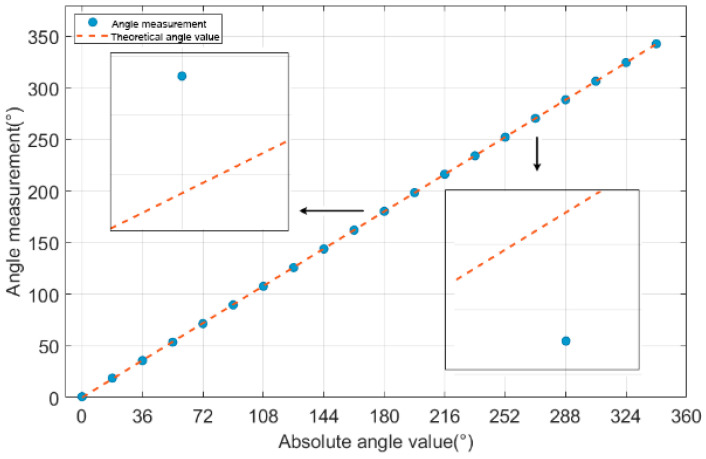
Results of angle measurement.

The absolute angle measurement results of the Hall angle sensor are shown in Figure 9, and its integral nonlinearity (INL) is less than ±0.71 deg. Reference [25] constructed an angle measurement system using Hall sensors and other discrete components. This system directly performed preamplification and ADC conversion on the voltage signals from the Hall sensors. There exists a considerable linearity error. The linearity error of the Hall sensor essentially originates from the combined effects of multiple factors, including material physical properties, geometric structure, circuit non-idealities, as well as external magnetic fields and mechanical conditions. Optimising the Hall voltage signal in the analogue front-end helps to further improve the INL performance. A comparison of key parameters is shown in Table 3. The TAD2141 product is an angle sensor designed based on TMR technology. A dynamic trimming technique is adopted in its digital circuit, which effectively reduces the integral nonlinearity error.

**Figure 10:**
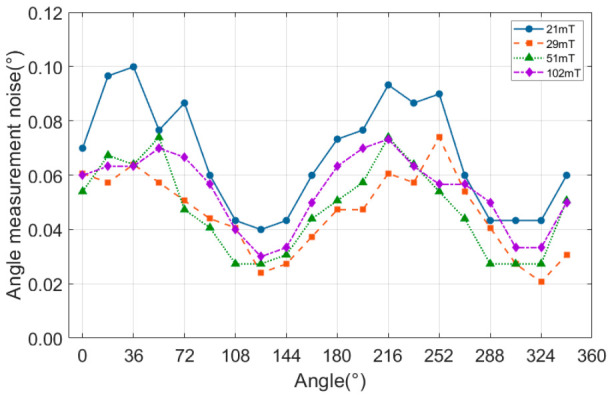
Noise test results under different magnetic field strengths.

The test results of angle measurement noise under different magnetic field strengths are shown in Figure 10. The minimum usable magnetic field strength is 30 mT. When the magnetic field strength is below 30 mT, the noise performance deteriorates significantly. On the one hand, when the magnetic field is small, the Hall voltage itself is weak, resulting in a significant decrease in the signal-to-noise ratio (SNR) and making the angle calculation results susceptible to noise interference. On the other hand, under a large magnetic field, the relative proportion of the interfering magnetic field is small; however, under a small magnetic field, its relative proportion increases significantly, which is equivalent to introducing additional noise when superimposed on the Hall signal. And, the saturation magnetic field strength is 100 mT.

**Figure 11:**
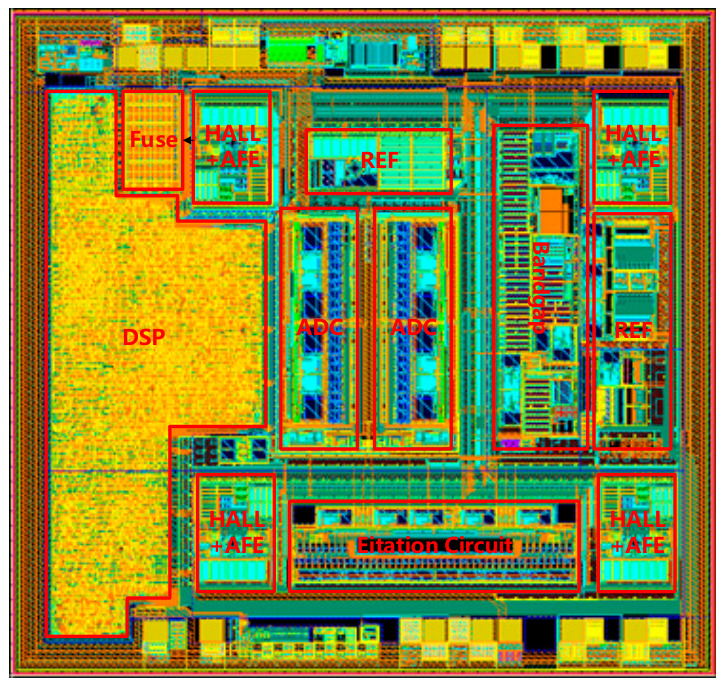
Chip layout.

The chip layout is illustrated in Figure 11, and the chip area is 2.8 mm × 2.6 mm. The Hall elements are arranged concentrically to maintain the required spatial configuration for angular sensing. Digital circuitry is implemented to perform CORDIC computation for real-time angle extraction. Additionally, an SPI digital communication interface is designed in this study, enabling direct readout of angular measurements by a microcontroller or FPGA.

## 4. Discussion

This paper presents a monolithically integrated angular position detection circuit, offering a low-cost and readily reproducible solution for angle measurement. The proposed design achieves effective suppression of process-gradient-induced offset voltage through concentrically symmetric Hall element placement, while the differential output architecture cancels common-mode temperature drift. Compared to single-Hall-element or asymmetrical layouts, the orthogonal configuration retains superior signal orthogonality in rotating magnetic fields, thereby reducing angle calculation errors.

The entire design is implemented in a standard CMOS process without requiring post-processing or specialised magnetic film integration, substantially reducing manufacturing costs while enhancing reliability. The on-chip integration of signal conditioning circuitry—including Hall sensor excitation, preamplification, and ADC—minimises reliance on external components, improving system immunity to interference and overall integration density. Furthermore, the embedded digital signal processing unit completes angle computation directly on-chip, eliminating the need for external processing.

Notably, the Hall sensors in this design require an external magnetic field strength exceeding 30 mT from a permanent magnet to achieve optimal angular detection performance. Additionally, precise alignment between the external magnet’s centre and the concentric centre of the four Hall elements is critical; any eccentricity degrades measurement accuracy. Relative to magnetoresistive sensors (AMR, GMR, and TMR), Hall sensors inherently exhibit higher baseline noise. The measured noise floor of 0.087° (3σ) in this design is primarily attributable to semiconductor fabrication process variations—including non-uniform N-well thickness and doping concentration inhomogeneities—which inevitably impact angular measurement precision. To address absolute zero-angle errors introduced during packaging due to chip misalignment, an eFuse trimming circuit is incorporated for calibration.

Compared to magnetoresistive sensor solutions, Hall-based sensors offer distinct advantages in integration density and cost, requiring neither MEMS fabrication processes nor system-in-package (SiP) assembly. Relative to optical encoders, the proposed solution provides smaller form factor, superior resistance to contamination and vibration, making it well-suited for industrial applications, humanoid robotics, and intelligent driving systems. However, for ultra-high-precision applications such as precision servo control requiring accuracy below 0.01°, further optimisation of temperature coefficient and long-term stability remains necessary.

Several aspects of this study warrant further investigation. First, silicon-based Hall sensors fabricated using CMOS N-well processes are inherently susceptible to temperature variations. Although the analogue front end incorporates architectures that suppress temperature drift, additional digital calibration algorithms could be implemented. On-chip temperature sensors combined with look-up-table-based compensation schemes could further extend the operating temperature range and improve angular measurement linearity [26].

Second, it should be noted that the present characterisation was conducted exclusively under static uniform magnetic field conditions. The dynamic performance of the angle sensor—particularly output propagation delay and measurement bandwidth under rapidly varying magnetic fields—requires further evaluation to determine its suitability for dynamic applications such as automotive electric power steering (EPS) systems [27,28].

## 5. Conclusions

This paper presents a single-chip angular measurement solution based on a four-Hall-element array, with high precision and miniaturisation as its core design objectives. Within a compact active area of 2.8 mm × 2.6 mm fabricated in a standard CMOS process, the proposed system monolithically integrates Hall sensors, a low-noise front end, a sigma-delta ADC, and digital signal processing units—realising a complete ‘magnetic-to-electrical-to-angular’ signal chain without requiring any external components. To address the inherent offset and temperature drift limitations of Hall devices, a current-domain vector summation architecture is introduced, effectively suppressing residual offset. Experimental characterisation demonstrates that the proposed sensor achieves an angular noise floor as low as 0.087° (3σ). This solution provides a reproducible and scalable technological pathway toward high-precision, low-cost, and miniaturised angular measurement.

## Figures and Tables

**Table 1:** Comparison of angle measurement noise.

	This Work	[24]	AS5040	TAD2141
Optimal Applied Magnetic Field Range	30–100 mT	100 mT	45–75 mT	20–80 mT
Maximum Angle Measurement Noise	0.066° (rms)	0.1° (rms)	0.12 °(rms)	0.092° (avg)

**Table 2:** Angular measurement noise under different temperature conditions.

Temperature	25 °C	45 °C	65 °C	85 °C	95 °C	105 °C
Maximum Angle Measurement Noise	0.040°	0.034°	0.051°	0.062°	0.079°	0.084°

**Table 3:** Comparison of integral nonlinearity.

	This Work	[25]	AS5040	TAD2141
INL (°)	±0.71	±1.263	±0.90	±0.30

## Data Availability

The overall research and development stage of this project has not yet been completed; thus, it is not appropriate to provide the original test data at present.

## Abbreviations

The following abbreviations are used in this manuscript:

CMOS	Complementary Metal Oxide Semiconductor
ADC	Analogue-to-Digital Converter
CORDIC	Coordinate Rotation Digital Computer
SPI	Serial Peripheral Interface
FPGA	Field-Programmable Gate Array
MEMS	Micro-Electro-Mechanical System
AMR	Anisotropic Magnetoresistance
GMR	Giant Magnetoresistance
TMR	Tunnel Magnetoresistance
